# Intraoperative Beat-to-Beat Pulse Transit Time (PTT) Monitoring via Non-Invasive Piezoelectric/Piezocapacitive Peripheral Sensors Can Predict Changes in Invasively Acquired Blood Pressure in High-Risk Surgical Patients

**DOI:** 10.3390/s23063304

**Published:** 2023-03-21

**Authors:** Michael Nordine, Marius Pille, Jan Kraemer, Christian Berger, Philipp Brandhorst, Philipp Kaeferstein, Roland Kopetsch, Niels Wessel, Ralf Felix Trauzeddel, Sascha Treskatsch

**Affiliations:** 1 Department of Anesthesiology and Intensive Care Medicine, Hindenburgdamm 30, Charité–Universitätsmedizin Berlin, Corporate Member of Freie Universität Berlin and Humboldt Universität zu Berlin, 12203 Berlin, Germany; michaelnordine@protonmail.com (M.N.);; 2 Berlin Institute of Health at Charité, Universitätsmedizin Berlin, Charitéplatz 1, 10117 Berlin, Germany; 3 Department of Physics, Humboldt University zu Berlin, 10115 Berlin, Germany; 4 SectorCon-Ingenieurgesellschaft mbH, 10553 Berlin, Germany; 5 Department of Human Medicine, MSB Medical School Berlin GmbH, 14197 Berlin, Germany

**Keywords:** piezoelectric, piezocapacitive, pulse transit time, non-invasive hemodynamics, intraoperative blood pressure, anesthesiology

## Abstract

Background: Non-invasive tracking of beat-to-beat pulse transit time (PTT) via piezoelectric/piezocapacitive sensors (PES/PCS) may expand perioperative hemodynamic monitoring. This study evaluated the ability for PTT via PES/PCS to correlate with systolic, diastolic, and mean invasive blood pressure (SBP_IBP_, DBP_IBP_, and MAP_IBP_, respectively) and to detect SBP_IBP_ fluctuations. Methods: PES/PCS and IBP measurements were performed in 20 patients undergoing abdominal, urological, and cardiac surgery. A Pearson’s correlation analysis (r) between 1/PTT and IBP was performed. The predictive ability of 1/PTT with changes in SBP_IBP_ was determined by area under the curve (reported as AUC, sensitivity, specificity). Results: Significant correlations between 1/PTT and SBP_IBP_ were found for PES (r = 0.64) and PCS (r = 0.55) (*p* < 0.01), as well as MAP_IBP_/DBP_IBP_ for PES (r = 0.6/0.55) and PCS (r = 0.5/0.45) (*p* < 0.05). A 7% decrease in 1/PTT_PES_ predicted a 30% SBP_IBP_ decrease (0.82, 0.76, 0.76), while a 5.6% increase predicted a 30% SBP_IBP_ increase (0.75, 0.7, 0.68). A 6.6% decrease in 1/PTT_PCS_ detected a 30% SBP_IBP_ decrease (0.81, 0.72, 0.8), while a 4.8% 1/PTT_PCS_ increase detected a 30% SBP_IBP_ increase (0.73, 0.64, 0.68). Conclusions: Non-invasive beat-to-beat PTT via PES/PCS demonstrated significant correlations with IBP and detected significant changes in SBP_IBP_. Thus, PES/PCS as a novel sensor technology may augment intraoperative hemodynamic monitoring during major surgery.

## 1. Introduction

Perioperative hemodynamic optimization, particularly optimal blood pressure (BP) management and early detection of BP fluctuations are critical for perioperative outcomes [1,2,3,4]. Beat-to-beat BP monitoring can be achieved by either invasive or non-invasive methods [5]. A critical requirement for such sensors is the ability to record reliable arterial pulse waves (PWs). From these PWs, the pulse transit time (PTT) can be extrapolated, which can serve as a surrogate BP marker.

Tracking PTT through PW analysis can be accomplished by vascular unloading techniques using optical or ultrasound-based sensors via pulse plethysmography (PPG). While these techniques have their respective advantages, they can be associated with patient discomfort, high cost, and potential problems with optical sensors such as light interference, as well as accuracy issues [6,7]. Other issues include the limited amount of clinically valid data generated from the perioperative period, where reliable, non-invasive solutions for continuous beat-to-beat BP tracking are needed.

An emerging sensor technology that can non-invasively track peripheral PWs is dual piezoelectric (PES)/piezocapacity (PCS) film sensors. PES/PCS sensors have been a mainstay in the industrial sector and are now being used as biomedical sensors [8]. These novel sensors function in the following manner: incoming PWs induce sensor deformation, resulting in electric depolarization proportional to pressure [9]. This depolarization is then converted into a readable arterial PW (signal transduction method) [10]. The piezocapacitive portion of the sensor converts this pressure change into a capacitance change (relative pressure change) [11], with a lower pressure sensitivity [12]. The piezoelectric section converts pressure signals directly into electrical signals with high-pressure sensitivity (absolute pressure change) [12]. Combined, this dual-sensor system can now detect both relative and absolute changes in pressure, providing two distinct waveforms. A comprehensive review of PES/PCS characteristics has been described previously [8,13].

However, there is a lack of data regarding the applicability of these novel PES/PCS to reliably track PTT in the perioperative period in high-risk surgical patients. It also remains unclear whether PTT obtained from these types of sensors could correlate with invasively acquired blood pressure (IBP) better than or equal to studies using other sensor technology. Another critical point of investigation is to determine if beat-to-beat PTT via PES/PCS could detect early-onset BP fluctuations. Therefore, this study aimed to deploy non-invasive PES/PCS to determine valid PW morphology (PW_PES_/PW_PCS_) and compare it with PW_IBP_. In addition, PTT via PES/PCS was evaluated to determine correlations with invasively acquired systolic, diastolic, and mean BP (SBP_IBP_, DBP_IBP_, MAP_IBP_, respectively), as well as their potential to predict intraoperative SBP_IBP_ fluctuations.

## 2. Methods

### 2.1. Study Design

This study was designed as a prospective observational study and was conducted at the Charité–Universitätsmedizin Berlin. All procedures involving human participants were in accordance with the ethical standards of the Institutional Research Committee and with the Declaration of Helsinki of 1964 and its subsequent amendments. The study was approved by the local ethics committee (EA1/155/17) and registered at ClinicalTrials.gov (NCT 03263988). Informed written consent was obtained from all patients enrolled in the study.

### 2.2. Study Inclusion and Exclusion Criteria

All adult patients admitted for elective major abdominal, urological, and cardiac (including cardiopulmonary bypass [CPB]) surgery under general anesthesia were screened for study inclusion. Exclusion criteria were defined as follows: American Society of Anesthesiologist (ASA) classification ≥ 4, pregnancy, atrial fibrillation or other serious cardiac arrythmias, presence of cardiac pacemakers, arteriovenous fistulas in the upper extremities, body mass index (BMI) > 35 kg/m^2^, inability to place the PES/PEC around the index finger, a difference in blood pressure measured between the two arms >12 mmHg, severe valvular disease, left ventricular ejection fraction (LVEF) < 35%, tricuspid annular plane systolic excursion (TAPSE) < 16 mm or the need for any type of left ventricular assist device or the inability to give informed consent.

### 2.3. Study Protocol

Upon arrival in the anesthesia induction area, standard monitoring was initiated, including a 3-lead electrocardiogram (ECG), pulse oximetry monitoring, and initial non-invasive oscillometric blood pressure monitoring. All patients underwent general anesthesia according to the local standard operating procedure (SOP). For patients undergoing major abdominal or urological surgery, anesthesia was induced with fentanyl (1–2 µg/kg), propofol (1–2 mg/kg) and cisatracurium (0.15 mg/kg). Anesthesia was maintained with sevoflurane or propofol with intermittent boluses of fentanyl and cisatracurium as needed at the discretion of the anesthesiologist. If clinically indicated, a peridural catheter was placed (TH 8–12), infusing a solution of 0.2% ropivacaine (6–8 mL/h). For patients undergoing cardiac surgery, induction was performed with propofol (1–2 mg/kg), sufentanil (0.1–0.5 µcg/kg) and cisatracurium (0.1 mg/kg). Prior to CPB, anesthesia was maintained with sevoflurane (approximately 1.0 MAC) and a continuous infusion of sufentanil (0.2–0.5 µg/kg/h) and on-CPB pump with propofol (6 mg/kg/h) for regulatory reasons. Ventilation was performed by pressure-controlled ventilation (PCV) with the goal of maintaining an end-tidal C0_2_ (etC0_2_) between 35 and 40 mmHg.

For IBP monitoring, an arterial catheter was placed in the left radial artery using the Seldinger technique and values were recorded on a beat-to-beat basis. General intraoperative hemodynamic management in all patients was aimed at maintaining mean arterial pressure (MAP) ≥ 65 mmHg via judicious administration of vasoactive medications or balanced intravenous crystalloid fluids.

PES/PEC sensors were attached to the patient’s right index finger after induction to reduce artifacts, and measurements began after signal quality was checked. Data from these sensors were collected from this point until the end of surgery.

**Figure 1:**
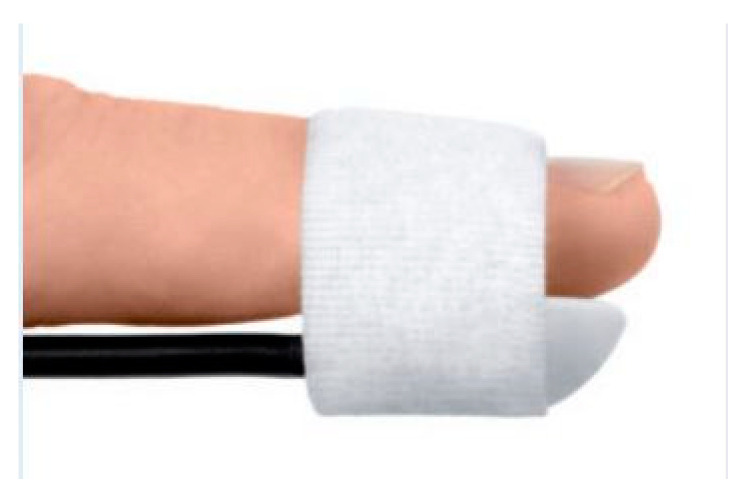
Example of dual PES/PCS sensor affixed to the index finger.

**Figure 2:**
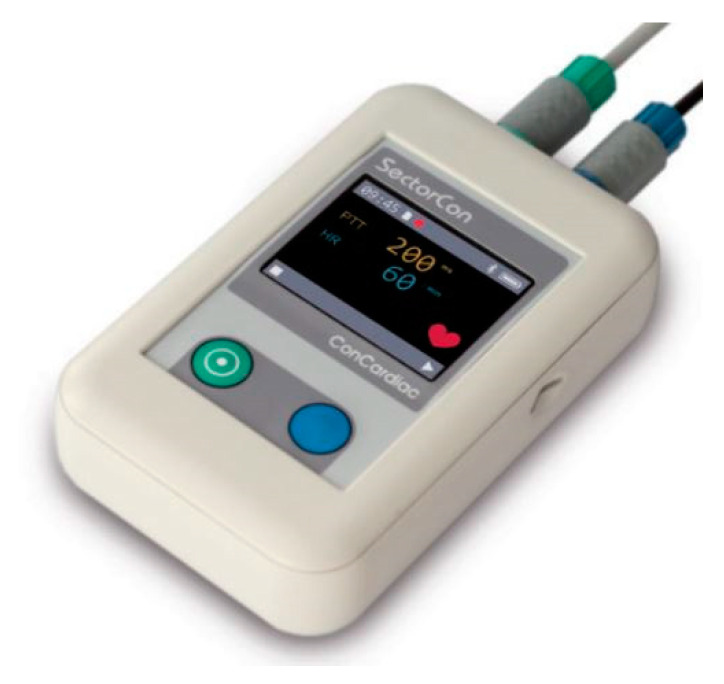
Data collection device for the PES/PCS system.

**Figure 3:**
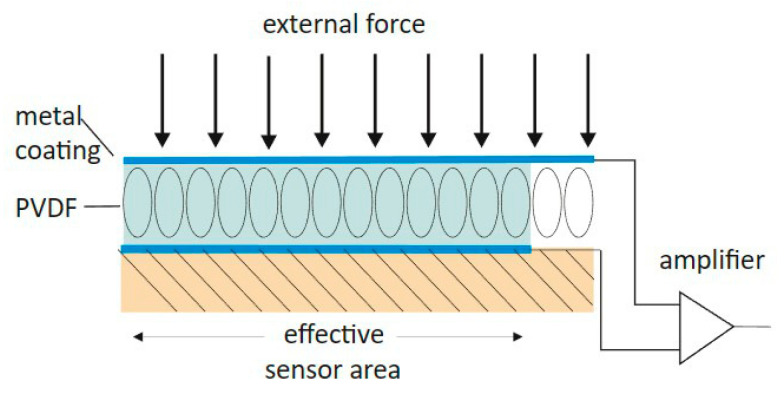
Geometric schematic of the dual PES/PCS sensor system. Described are the main components that track and relay the signals from an incoming force. PVDF = polyvinylidene fluoride.

The PES/PEC sensor system used was developed and manufactured by SectorCon (SectorCon Ingenieurgesellschaft mbH, Berlin, Germany). The dual sensors were composed of a piezoelectric and a piezocapacitive section, both relaying 2 unique PWs with distinct characteristics (low/high frequency tracking, absolute/relative pressure changes). They could measure a pressure range between −5 and 50 kPa at a sampling rate of 250 Hz. PWs and PTT were recorded and captured using a handheld device developed by the same company, including a separate 3-lead ECG cable as reference. PW amplitude was reported in arbitrary units (a.u.) and PTT (measured from the beginning of the R-peak of the ECG) was defined as milliseconds. A total of 2 unique PWs and 2 PTTs were recorded and analyzed on a beat-to-beat basis. The data were converted to an ASCII file for offline analysis. An example of a PES/PEC sensor is shown in Figure 1, and the data acquisition device is shown in Figure 2. The schematic geometric structure of the PES/PCS is displayed in Figure 3.

### 2.4. Data Analysis

PW_PES_/PW_PCS_ were analyzed in the following steps to determine the frequency of reliable PW morphology:

(1) Detection of R-peaks in the ECG using a Hilbert transformation-based algorithm [14] which were used as the defining point for the start of a new beat-to-beat PW;

(2) Calculation of the mean PW from the non-invasive sensors and the correlation p of each beat-to-beat PW to the mean PW. The area under the PW was defined as its magnitude (mag). A PW was marked as an outlier if one of the following two conditions was not met:

- ρ > 0.85;
- The absolute relative difference in magnitude from the mean PW (Δmag) was less than 0.5;

(3) PTT was defined as the time interval from the R-peak to the steepest rise in PW;

(4) This procedure resulted in a PTT time series with missing values that were interpolated using the 4th order weighted essentially non-oscillatory (WENO4) technique [15].

**Figure 4:**
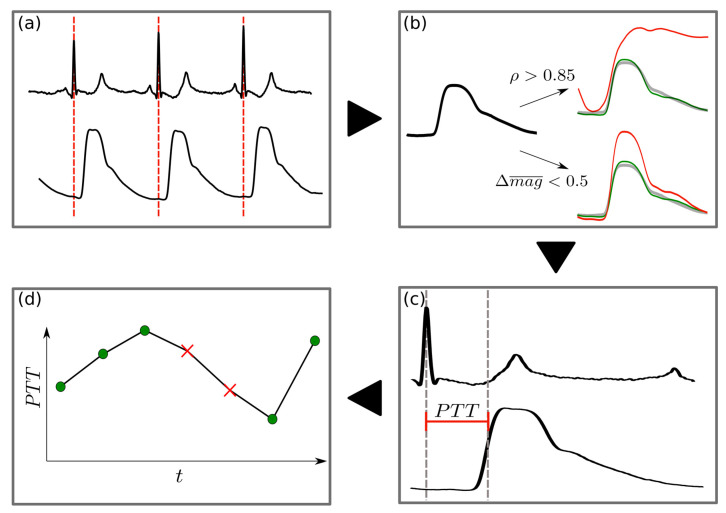
Flowchart of the PW detection pre-processing: (**a**) The R-peak in the ECG defines the PW onset. (**b**) The mean PW is identified as a robust template for physiologically plausible PWs in terms of morphology and magnitude. Each PW is then compared with a patient-specific template (green PW) for automatic deselection of non-optimal PWs (e.g., due to motion noise). A PW is rejected if either its Pearson correlation with the template is less than 0.85 or if the difference in normalized magnitude (i.e., area under the curve [AUC]), is greater than 0.5 (red PW). All remaining PWs are defined as valid. (**c**) For all valid PWs, PTT is calculated as the time from R-peak to the steepest rise in the PW. (**d**) For R-peaks where a PW is rejected, PTT values are inputted using a 4th order interpolation technique, WENO4. This interpolation is justified by the fact that 95% of all gaps in the PTT signal are less than 10 beats in length.

**Figure 5:**
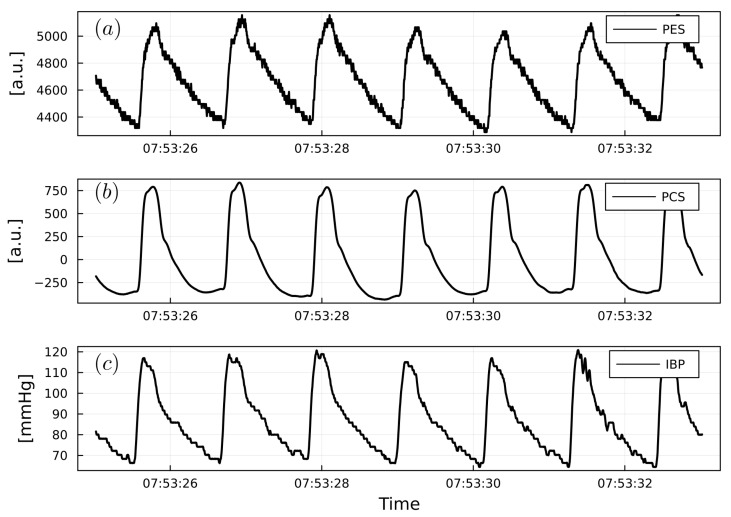
(**a**) Example of PW_PES_. (**b**) Example of PW_PCS_. (**c**) Example of PW_IBP_ from a cardiac surgery patient. PW_PES_ and PW_PCS_ are reported as arbitrary units (a.u.). PW_IBP_ is reported as mmHg.

**Figure 6:**
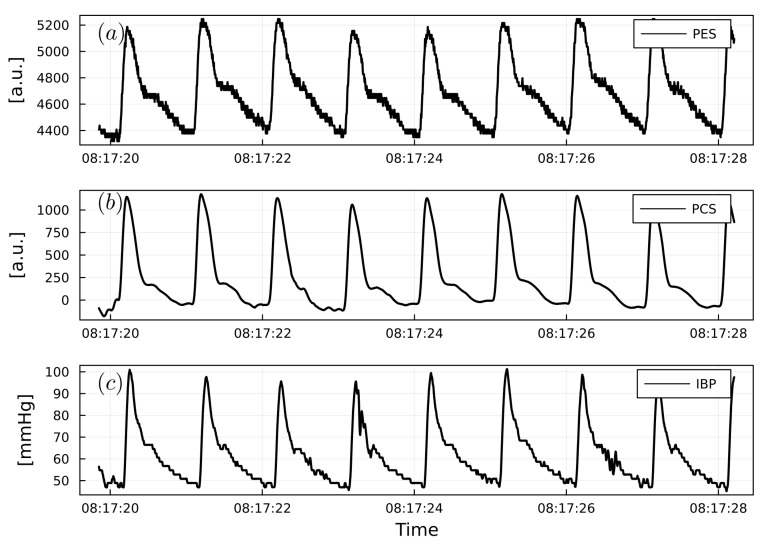
(**a**) Example of PW_PES_. (**b**) Example of PW_PCS_. (**c**) Example of PW_IBP_ from a urological surgery patient. PW_PES_ and PW_PCS_ are reported as arbitrary units (a.u.). PW_IBP_ is reported as mmHg.

This was performed for each patient and for all non-invasive and invasive PWs (Figure 4). Two patient specific examples of PW analysis are shown in Figure 5 and Figure 6.

### 2.5. Statistical Analysis

For beat-to-beat correlations between IBP and 1/PTT (inverse of PTT) via PES/PCS, Pearson’s correlation coefficient tests (r) were performed. An area under the curve receiver operating curve (AUROC) was used to decipher the ability of PTT to detect fluctuations in intraoperative IBP. Significant fluctuations were defined as SBP_IBP_ either increasing or decreasing by more than 30% compared with the mean BP of each patient. Because absolute PTT is specific to each individual, 1/PTT was converted to relative changes from baseline. The performance of the 1/PTT predictor was adjusted by AUROC analysis to determine the optimal cutoff based on maximizing sensitivity and specificity. PW reliability and correlation data were reported as median and interquartile range (IQR). Patient data were reported as mean with ± standard deviation (SD). Statistical analysis was performed using the Julia programming language version 1.6.3 with the StatsBase library [16].

## 3. Results

After completion of the study, data from 20 consecutive patients were analyzed. Patient characteristics, type of surgery and anesthesia are listed in Table 1.

### 3.1. PW Reliability Detection

At study completion, 92% (7.6) PW_PES_ and 93% (6.9) of all PW_PCS_ recorded during the inoperative period met the reliability criteria as described in the methods section. In comparison, 97% (2.2) of all PW_IBP_ met the reliability criteria.

### 3.2. 1/PTT and IBP: Correlations and Predictive Capabilities

**Figure 7:**
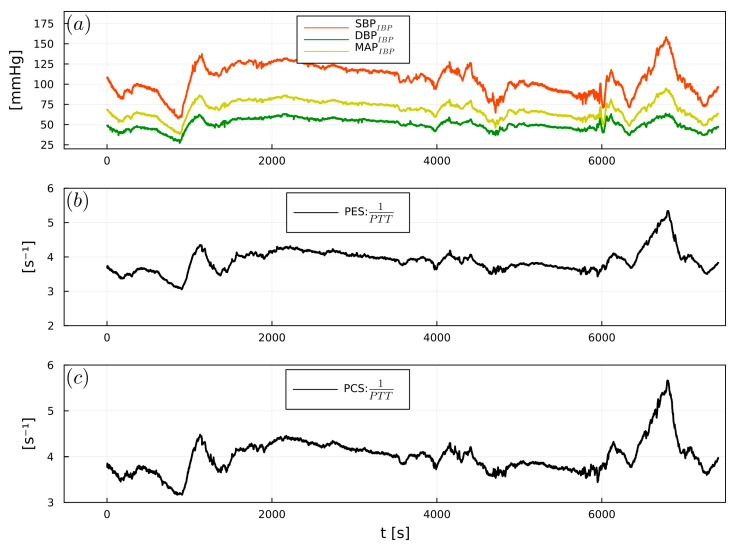
Example of (**a**) intraoperative SBP_IBP_ (red), DBP_IBP_ (green), and MAP_IBP_ (yellow), (**b**) 1/PTT_PES_, (**c**) 1/PTT_PCS_, from a cardiac surgery patient.

**Figure 8:**
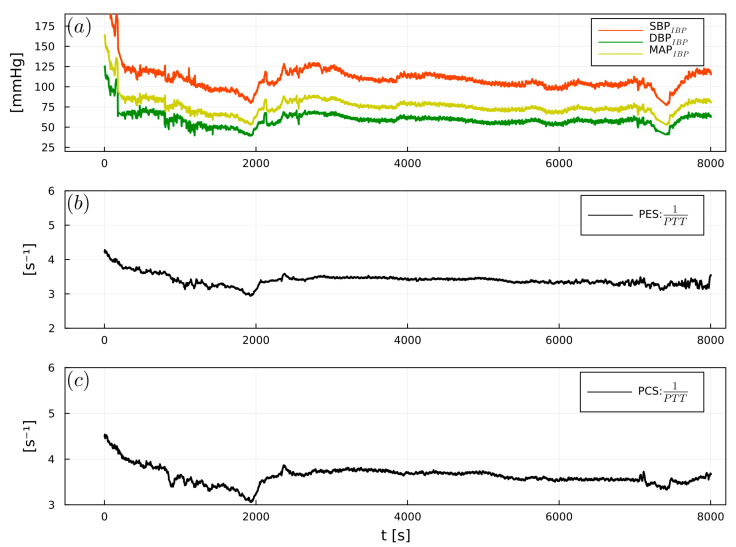
Example of (**a**) intraoperative SBP_IBP_ (red), DBP_IBP_ (green), and MAP_IBP_ (yellow), (**b**) 1/PTT_PES_, (**c**) 1/PTT_PCS_, from an abdominal surgery patient.

**Figure 9:**
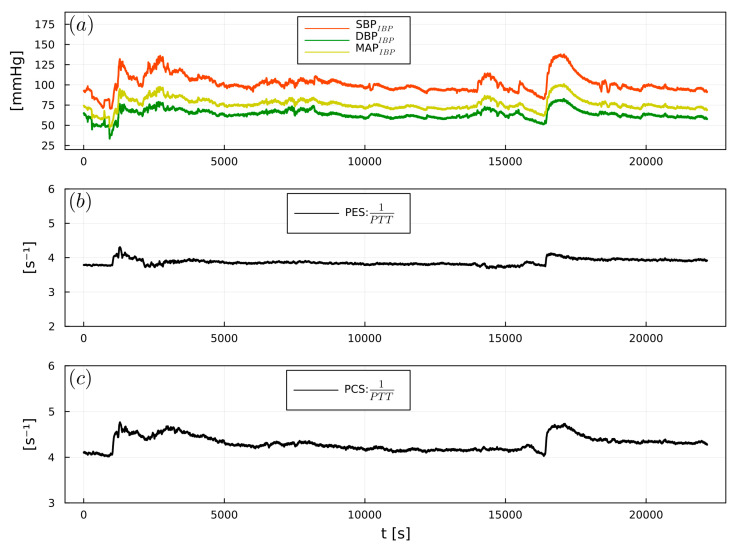
Example of (**a**) intraoperative SBP_IBP_ (red), DBP_IBP_ (green), and MAP_IBP_ (yellow), (**b**) 1/PTT_PES_, (**c**) 1/PTT_PCS_, from a urological surgery patient.

**Figure 10:**
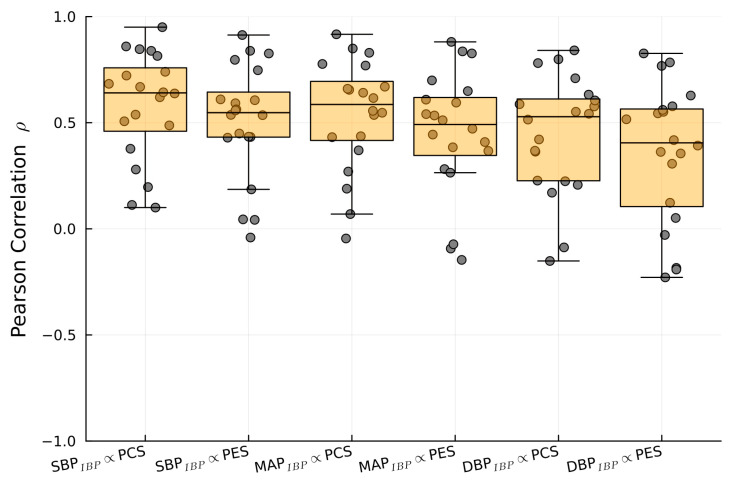
Boxplot of the Pearson correlation between SBP_IBP_, MAP_IBP_, and DBP_IBP_ with 1/PTT_PES_ and 1/PTT_PCS_.

1/PTT showed significant correlations between PES and SBP_IBP_ (r = 0.64), DBP_IBP_ (r = 0.55), and MAP_IBP_ (r = 0.6). PCS exhibited significant correlations with SBP_IBP_ (r = 0.55), DBP_IBP_ (r = 0.45), and MAP_IBP_ (r = 0.5) (Table 2). Figure 7, Figure 8 and Figure 9 show examples of the intraoperative course of 1/PTT with both PES/PEC sensors overlaid with SBP_IBP_, DBP_IBP_, and MAP_IBP_ from abdominal, urological, and cardiac surgery. Box plots highlighting the correlation data are shown in Figure 10.

**Figure 11:**
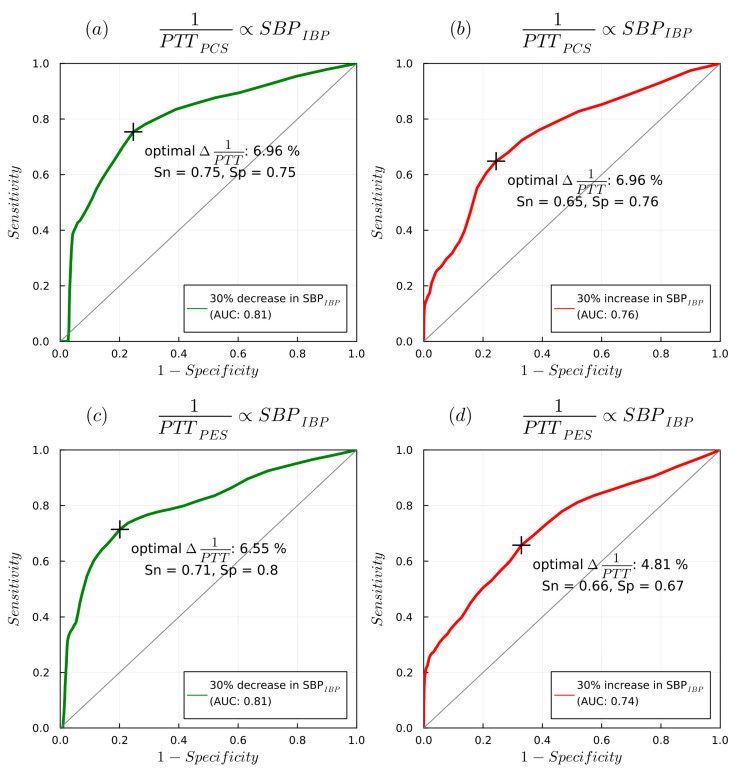
AUROC curves detailing 1/PTT changes detected with PES/PCS sensors with changes in SBP_IBP_: (**a**) delta of 1/PTT_PCS_ with decrease in SBP_IBP_, (**b**) delta of 1/PTT_PCS_ with increase in SBP_IBP_, (**c**) delta of 1/PTT_PES_ with decrease in SBP_IBP_, (**d**) delta of 1/PTT_PCS_ with increase in SBP_IBP_.

AUROC curve analysis (Figure 11) showed that a 7% decrease in 1/PTT_PES_ could predict a 30% decrease in SBP_IBP_ (AUC 0.82, sensitivity 0.76, specificity 0.76), whereas a 5.6% increase predicted a 30% increase in SBP_IBP_ (AUC 0.75 sensitivity 0.7 specificity 0.68). A 6.6% decrease in 1/PTT_PCS_ could detect a 30% decrease in SBP_IBP_ (AUC 0.81, sensitivity 0.72, specificity 0.8), while a 4.8% increase in 1/PTT_PCS_ could predict a 30% increase in SBP_IBP_ (AUC 0.73, sensitivity 0.64, specificity 0.68).

## 4. Discussion

This study demonstrated that novel PES/PEC sensors have a >90% reliable intraoperative PW detection rate in patients undergoing major abdominal, urological, and cardiac surgery. Significant correlations were also observed between intraoperative SBP_IBP_ and both 1/PTT_PES_ and 1/PTT_PCS_, while significant, albeit lower correlations were found for DBP_IBP_ and MAP_IBP_, consistent with previous studies [17]. In addition, an approximate 7% change in 1/PTT_PES_ and 1/PTT_PCS_ sensors could predict a 30% change in intraoperative SBP_IBP_ fluctuations. The only other comparable study of this type found that a 15% change in PTT could predict a 30% change in SBP_IBP_ via PPG sensors [17]. Thus, the results of this monocentric study highlight the potential of beat-to-beat PTT tracking via PES/PCS as an innovative non-invasive sensor technology that may enhance future intraoperative hemodynamic monitoring.

### 4.1. PW Analysis and Prior PES/PEC Research

A critical requirement for PTT determination is the ability to reliably detect incoming PWs. In this context, the PW is a complex physiological signal containing multiple components of the cardiac cycle, that reverberates throughout the body, and is tracked via sensors from the distal extremities. The time delay (measured from the onset of the R-peak of the electrocardiogram [ECG]) required for a PW to travel from the left ventricle to any (distal) anatomical location is defined as the PTT, which includes the pre-ejection period (PEP) and pulse arrival time (PAT) [18,19]. PTT has been found to have strong correlations with systemic BP fluctuations [20], and has previously been studied in clinical settings [21]. Furthermore, PTT, thus, may reflect variations in PEP and central blood volume and is useful for early detection of non-hypotensive progressive central hypovolemia [22]. Given its physiological background, PTT may present an ideal surrogate biosignal for peripheral non-invasive BP tracking [19]. However, the use of PTT as a surrogate BP marker may be complicated by the PEP component, as PEP is strongly influenced by mechanical and electrical cardiac factors, which may negate BP correlations [23]. Additionally, PEP is highly inter-individual dependent and is strongly influenced by sympathetic tone during mental stress, which may have implications when used in a general perioperative setting [24]. Previous work by Finnegan et al. found that during vasoactive administration, PTT showed greater correlations with BP than PAT [18], a finding highly relevant to the use of PTT in the perioperative setting, where the use of vasoactive medications is quite common.

Previous studies using piezo-based sensors have been able to reliably detect PWs in healthy volunteers [25,26], volunteers with arrythmias [23], and hypertensive patients [12]. In a recent study, the use of novel piezoelectric sensors resulted in a PW detection rate of 98% from the radial artery [27]. In a clinical setting, Clemente et al. also demonstrated a reliable beat-to-beat PW detection rate in a small cohort of ICU patients [28]. All of these studies demonstrated a reliable piezosensor-based PW detection rate of >90%, which was also confirmed in our study. Not only did the PES/PCS system detect a >90% PW_PES_/PW_PCS_ rate in our study but was able to do so in a rigorous perioperative environment from high-risk surgical patients, where rapid changes in BP are common.

Disruptive perioperative factors such as electrocauterization, extreme BP fluctuations, positional changes, intra-abdominal pressure, and changes in vascular tone due to bleeding, pain and vasoactive medications contribute to a challenging environment for testing novel non-invasive sensors. However, PES_PW_/PCS_PW_ validity was found to be >90%, compared with a 97% validity for PW_IBP_. Despite these confounding factors, PES/PSC were able to decipher a high rate of beat-to-beat PTT for all valid PWs, which has also been demonstrated in ambulatory hypertensive patients [29]. Another technical challenge of the PES/PCS was the correct amount of external pressure needed to obtain an optimal PW measurement. The optimal contact pressure for piezoelectric sensors is in the range of 1.6–2 Newtons [30]. The advantage of the novel PES/PCS demonstrated in this study was the ability of the PCS portion to register the amount of pressure applied, thereby acting as a PW quality control mechanism. This is a feature that PPG sensors do not include. Future studies comparing PES/PCS with PPG based sensors to determine PW reliability, correlations, and predictive capabilities in the perioperative setting are, thus, needed to verify these findings.

### 4.2. PTT: Correlation and Predictive Capabilities

While no study has investigated the correlations between piezosensor-based PTT and IBP, as well as the ability of piezosensor-derived PTT to predict IBP fluctuations in the perioperative environment, there are a handful of comparable PPG studies of this nature. The correlation between non-invasively acquired PTT measured from the delay between the R-peak of the ECG and the peak of a PPG signal with BP has been well established in theoretical models [31]. Non-invasively acquired PTT has also been found to have a significant correlation with cuff-based SBP in healthy volunteers as well [32], although other studies have refuted this correlation [33]. Regarding the correlations between non-invasive PTT and BP, PTT shows a good correlation with SBP, while correlations between MAP and DBP are inconsistent [18,23,34]. Two studies have provided evidence that piezosensor-derived PTT shows a significant correlation and accuracy with PTT obtained from PPG devices [9,26]. One study demonstrated a significant correlation between PTT and SBP during the administration of vasoactive medications in an experimental setting, which is highly relevant for the perioperative environment, as vasoactive medications are often administered to stabilize hemodynamic status [34]. A strong significant correlation between PTT and SBP_IBP_ was found in healthy volunteers undergoing dobutamine infusion, while a lower yet significant correlation between PTT and DBP_IBP_ was also determined [35]. In a retrospective analysis of a large database of surgical patients, PPG-derived PTT showed a very weak correlation with invasively and non-invasively acquired BP [36]. Finally, previous experimental work found that PTT showed superior IBP correlations compared with PAT during significant IBP fluctuations, concluding that PTT is a far more robust surrogate parameter for IBP tracking than PAT [35]. Similar findings have also been reported with PTT showing superior IBP correlations to PAT [32]. The results of this study further solidify the correlations between IBP and PTT measured in a handful of studies. The major finding of this study was the significant correlations found in high-risk surgical patients experiencing IBP fluctuations.

The only directly comparable study examining non-invasively acquired PTT in hypertensive renal transplant patients in the perioperative setting, found a good correlation between PTT and SBP_IBP_, while moderate correlations were found with MAP_IBP_ and DBP_IBP_ [17]. Its main difference with our study was the technique used to measure PTT (PCS/PES vs. PPG), and the time period of measurement (intraoperative vs. induction). Kim et al. also found a higher level of correlation for SBP (r ≥ 0.8), MAP (r = 0.8), and DBP (r = 0.6), than the correlations demonstrated by PES/PCS in this study. This is likely due to the time in which these correlations were recorded (induction period vs. post-induction period), where confounding intraoperative factors are minimal. Despite these differences, our study confirmed this correlation trend.

Another clinical study tracking PTT during spinal anesthesia showed a good correlation between PTT and non-invasively measured MAP, while no information was given on correlations between SBP and DBP [37]. A retrospective analysis of >500 patients who experienced a rapid decrease in SBP in a non-perioperative setting showed a correlation between PTT and SBP of r = −0.91 [38]. The significant correlation between PTT and SBP was attributed to SBP being influenced by both cardiac activity and vascular tone [38]. In the same study, the authors found no correlation between PTT and DBP and MAP. The reason for this is that both DBP and MAP are indicators of vascular stiffness, which diminishes the correlation with PTT. The reason stronger correlations are found between PTT and SBP may be due to pressure and velocity factors, as well as the influence of the incoming pressure wave registered by hemodynamic sensors. The systolic cycle involves the forward propulsion of blood (velocity) which exerts a pressure gradient (pressure) that accumulates as a pressure wave, in contrast to DBP and MAP [39]. However, our study found moderate correlations between PTT with MAP_IBP_ and DBP_IBP_, which supported the findings of Kim et al., and refuted the findings of Payne et al. These significant correlations may be due to the residual effects of the pulse reflection wave during DBP, thereby influencing MAP [17].

Our patient cohort consisted of high-risk patients with comorbidities that may have affected PTT measurements. The presence of cardiovascular comorbidities may impact the relationship between PTT and systemic BP, as the structural changes in the myocardium and vascular architecture may alter PW propagation [40]. Other clinical studies have found that the relationship between PTT and systemic BP is not affected in patients with hypertension [17,41]. Another study examining hemodialysis patients undergoing simulated fluid shifts via lower body negative pressure (LBNP), found that distally measured PTT showed a good correlation during acute decreases in systemic BP [41]. Interestingly, despite the presence of high-risk patients with comorbidities, significant correlations were found between PTT and IBP, suggesting that augmenting hemodynamic monitoring with PES/PCS is feasible, even in patients with pre-existing cardiac dysfunction.

The ability of PTT to predict significant intraoperative BP fluctuations is a major advantage of this particular biosignal. There are a handful of clinical studies that highlight the potential of PTT to detect significant BP fluctuations. The most relevant study by Kim et al. found that a 15% change in 1/PTT could predict a 30% change in systolic blood pressure during induction of anesthesia [17]. During obstetric spinal anesthesia, beat-to-beat changes in PTT could detect significant blood pressure changes in normotensive and hypertensive women [37,42]. Our study supported these findings, where a lower 1/PTT change (approximately 7%) could detect 30% changes in IBP, compared with 15%. This may be due to differences in sensor technology and suggests the piezoelectric sensors may be more sensitive to subtle changes in peripheral vascular tone than PPG sensors. The 30% cutoff showed the highest sensitivity and specificity, while reducing this cutoff to 10–20% changes in SBP significantly reduced the sensitivity and specificity. Other studies have found a PTT cutoff of 15% to detect a >30% change in IBP [17], and a 20% change in intraoperative PTT could detect 10% changes in oscillometric MAP in women undergoing cesarean section [42]. Significant fluctuations in intraoperative BP, particularly >30% decreases in SBP can lead to critical reductions in perfusion to myocardial tissue and renal tissues, resulting in organ damage [43,44], and an increase in postoperative mortality [45] and morbidity [46]. Therefore, a >30% change in BP appears to be a clinically relevant cut-off point. Previous results have shown that PTT has a higher propensity than RR interval to predict autonomic responses to nociceptive stimulation and variations in depth of anesthesia [47]. PTT has also been shown to reliably indicate an effective axillary block via the loss of vasomotor tone. This was indicated by an increase of 12 ms 3 min after block with a sensitivity of 87% and specificity of 71% [48]. The results of our study suggested that smaller percentage changes in PTT may provide predictive information about BP fluctuations, but the sensitivity is dependent on the BP cut-off. Further studies are needed to determine the ideal cut-off BP.

The ability of PTT to serve as an early indicator of systemic BP fluctuations has shown promise in theoretical models [49], however there are only a handful of monocentric clinical trials to support this. All the above studies used PTT recorded by PPG sensors. Although both use different modalities of PW detection, both are comparable in terms of reliability. Recent non-clinical research has shown that both PPG and piezosensors are both equivalent in deciphering PWM and PTT from distal extremities [50]. Thus, the results of this study highlight the ability of PTT via PES/PCS to detect significant SBP_IBP_, DBP_IBP_, and MAP_IBP_ correlations, as well as the ability of these sensors to track intraoperative BP fluctuations. Detecting early onset BP fluctuations is a valuable tool for the anesthesiologist, however the ability to determine if an intervention is successful in correcting these fluctuations with PPG or PES/PCS warrants further investigation A recent report examining PTT via PPG found that PTT has a poor ability to determine fluid responsiveness in patients undergoing major abdominal surgery [51]. Thus, PTT may be useful in identifying impending BP fluctuations, but may not be useful to guide optimizing BP therapy.

### 4.3. Limitations

A limitation of the study was studying a small heterogeneous group of surgical patients receiving either inhalation (sevoflurane) or intravenous (propofol) anesthesia. While it is theorized that propofol may lead to greater peripheral arterial distension, no significant differences between propofol and sevoflurane-based anesthesia on PTT have been found [52]. The majority of patients in our cohort received vasopressor therapy and volume administration during surgery. Vasopressor therapy directly increases systemic vascular resistance, which has been shown to have no effect on overall PTT [34]. The effects of anesthetic interventions on PTT, PTT correlations and predictive ability in this study may or may not have influenced our results. Further studies investigating the effects of vasopressor and volume therapy on PTT are needed. Another limitation was the omission of PTT measurements during induction of anesthesia, as well as the lack of concurrent measurement with PPG, which could have been used to create a Bland–Altmann plot analysis. While both sensor techniques can record PW/PTT, the methodology is fundamentally different (pressure vs. infrared). Previous non-clinical studies have shown that both techniques are similar in terms of tracking physiological parameters via finger probe [53,54,55,56]. Another limitation is the sole use of PTT itself, instead of PEP or PAT. All three signals indicate distinct characteristics of the PW. PEP is strongly influenced by sympathetic tone [24], and the use of PEP may have altered the correlation results presented here. For the purpose of this proof-of-concept study, we chose to use the R-wave of the ECG as a starting point. Another reason for choosing PTT over PAT and PEP is that our study aimed to assess changes over a longer period of time as opposed to smaller and more rapid changes, for which PTT is better suited than PAT and PEP [57]. Furthermore, PTT was used so as to be comparable with other studies in the perioperative setting [17,37,42]. However, future studies should incorporate sensors that can distinguish the difference between PEP, PAT, and PTT to provide comprehensive data from the entire cardiac cycle that may be relevant for clinical scenarios. Finally, a statistically significant correlation between 1/PTT and IBP may not be suitable for individual prediction of BP values, but it may serve as a prerequisite for making such predictions possible.

### 4.4. Summary and Future Applications

This study used novel PES/PCS to track intraoperative beat-to-beat PWs and PTT in a heterogeneous cohort of patients undergoing major surgery. The results demonstrated a > 90% beat-to-beat PW reliability detection rate. PTT from these sensors showed a higher significant correlation with SBP_IBP_ on a beat-to-beat basis than DBP_IBP_ and MAP_IBP_. Finally, PTT from PES/PCS demonstrated the ability to detect early onset SBP_IBP_ fluctuations with a high degree of sensitivity and specificity, providing critically important hemodynamic information. The major advantage of PES/PCS sensors, over PPG technology, is the ability to use these sensors at sites other than the finger, as a PW can be detected with the appropriate applied pressure. Recent research has demonstrated the feasibility of using PES/PCS sensors to record continuous heart tones [58], and to decipher PWs from the neck and wrist [59]. In addition, the dual sensor components allow precise tracking of the PW using both relative pressure and absolute sensor contact pressure measurements. Prior research has only focused on relative pressure signals, which does not allow for verification that the sensor is properly seated with adequate contact pressure. Future studies will involve the use of these sensors on different areas of the body to assess a multi-dimensional level of hemodynamic status, as opposed to being regulated only on the distal extremities.

## Figures and Tables

**Table 1:** Patient demographics, biometrics, comorbidities, type of surgery, length of surgery, and inoperative vasoactive medications. Values are reported as numbers or mean ± standard deviation (SD).

	Patients’ Characteristics
12/8	Gender (F/M) (n)
62 ± 11.5	Age (years)
170 ± 0.15	Height (cm)
78 ± 18	Weight (kg)
25 ± 8.2	BMI (kg/m^2^)
1.93 ± 0.3	Body surface area (BSA) (m^2^)
	ASA classification (n)
10	II
10	III
	Comorbidities (n)
10	Arterial hypertension
5	Coronary artery disease
4	Diabetes mellitus
2	Heart failure
2	Chronic kidney disease
2	Hypothyroidism
2	Asthma bronchiale
1	Chronic obstructive pulmonary disease
	Type of surgery (n)
7	Cardiac (including CPB)
7	Abdominal (e.g., Whipple, pancreatic/intestinal/hepatic resection)
6	Urological (e.g., cystectomy)
	Average duration of surgery (min)
213 ± 30	Cardiac
214 ± 110	Abdominal
323 ± 23	Urological
	Intraoperative vasoactive medications
17	Norepinephrine (infusion)
2	Dobutamine (infusion)
2	Enoximone (infusion)
14	Caffeine/Theodrenaline (bolus)
6	Atropine (bolus)

**Table 2:** Correlation between 1/PTT via PES/PCS and SBP_IBP_, DBP_IBP_, and MAP_IBP_. Pearson correlation coefficient (r) and IQR are reported.

Sources Compared	Pearson’s Correlation (Median and IQR)
BP_IBP_ vs. 1/PTT_PES_	0.64 (0.32)
SBP_IBP_ vs. 1//PTT_PCS_	0.55 (0.28)
DBP_IBP_ vs. 1/PTT_PES_	0.55 (0.36)
DBP_IBP_ vs. 1/PTT_PCS_	0.45 (0.40)
MAP_IBP_ vs. 1/PTT_PES_	0.6 (0.31)
MAP_IBP_ vs. 1/PTT_PCS_	0.5 (0.27)

## Data Availability

The data presented in this study are available on request from the corresponding author (ST). The data are not publicly available due to patient privacy regulations and intellectual property reasons.
